# Structural Property Study for GeSn Thin Films

**DOI:** 10.3390/ma13163645

**Published:** 2020-08-17

**Authors:** Liyao Zhang, Yuxin Song, Nils von den Driesch, Zhenpu Zhang, Dan Buca, Detlev Grützmacher, Shumin Wang

**Affiliations:** 1 Department of Physics, University of Shanghai for Science and Technology, Shanghai 200093, China; 2 State Key Laboratory of Functional Materials for Informatics, Shanghai Institute of Microsystem and Information Technology, Shanghai 200050, China; songyuxin@gmail.com (Y.S.); zhp_zero@163.com (Z.Z.); 3 Jülich Aachen Research Alliance (JARA)-Institute Green IT, RWTH Aachen, 52074 Aachen, Germany; n.von.den.driesch@fz-juelich.de (N.v.d.D.); d.gruetzmacher@fz-juelich.de (D.G.); 4 Peter Grünberg Institute 9 (PGI-9) and JARA-Fundamentals of Future Information Technologies (JARA-FIT), Forschungszentrum Jülich, 52425 Jülich, Germany; d.m.buca@fz-juelich.de; 5 Department of Microtechnology and Nanoscience, Chalmers University of Technology, 41296 Goteborg, Sweden

**Keywords:** GeSn, structural property, XRD

## Abstract

The structural properties of GeSn thin films with different Sn concentrations and thicknesses grown on Ge (001) by molecular beam epitaxy (MBE) and on Ge-buffered Si (001) wafers by chemical vapor deposition (CVD) were analyzed through high resolution X-ray diffraction and cross-sectional transmission electron microscopy. Two-dimensional reciprocal space maps around the asymmetric (224) reflection were collected by X-ray diffraction for both the whole structures and the GeSn epilayers. The broadenings of the features of the GeSn epilayers with different relaxations in the *ω* direction, along the *ω*-2*θ* direction and parallel to the surface were investigated. The dislocations were identified by transmission electron microscopy. Threading dislocations were found in MBE grown GeSn layers, but not in the CVD grown ones. The point defects and dislocations were two possible reasons for the poor optical properties in the GeSn alloys grown by MBE.

## 1. Introduction

Since the first planar silicon (Si) transistor was invented in 1959 [1], the Si electronic industry has developed prosperously. According to the Moore’s law the number of components on integrated circuits (ICs) has doubled every 18 to 24 months for the last 50 years [1]. However, the continuous scaling of the transistors has led to several problems, such as signal delays, higher power consumption, quantum limitations, etc. Moreover, without new technologies, the shrinking of the transistors will stop around 2021 [2]. Si photonics, which utilizes photons instead of electrons for information transmission and processing, is one of the promising solutions for these problems and shows a bright future [3,4].

It is difficult to fabricate light sources using Si due to its indirect bandgap nature. The light sources used in the commercial Si based circuits are made of group III-V materials, and then bonded to the circuits at present [5]. However, the bonding technology hinders the future large-scale integration [3]. Monolithic light sources compatible with the complementary metal-oxide-semiconductor (CMOS) processing platform are in urgent need. Ge is also an indirect bandgap material, but the energy difference between its Г and L valley is only 140 meV [6], much smaller than that of Si. It is found that incorporation of Sn in Ge can reduce the energy of both the Г and the L valleys in the conduction band, while the Г valley goes downward faster. Eventually, the Ge_1-x_Sn_x_ alloy becomes a direct bandgap semiconductor with the Г valley lower than the L valley, when the Sn concentration is above about 8% [7]. When tensile strain is applied on Ge, a similar transformation of the band structure in the conduction band can happen [7]. However, devices based on tensile-strained Ge require complex processes, such as integration of Si_3_N_4_ stressor layers [8], or selective wet under-etching [9]. Moreover, GeSn is predicted to own high carrier mobility [10], making it a potential candidate for fabricating both electronic and optoelectronic devices integrated on the Si platform. GeSn p-MOSFETs on Si [11,12,13] and GeSn lasers [7,14,15,16,17] are already successfully realized. Especially, the successful demonstration of the GeSn lasers has drawn significant attention and confidence on the realization of CMOS technology compatible light sources monolithically integrated on Si platform.

The electrical property and (or) optical property of the as grown materials are critical for the performance of devices, both of which are highly related to the structural properties. For GeSn alloys, Sn incorporation is the largest growth challenge, due to the large miscibility gap of the Ge-Sn binary system [18] and the large lattice mismatch between Ge and α-Sn [7]. Molecular beam epitaxy (MBE) and chemical vapor deposition (CVD) are the main methods for the growth of GeSn alloys [19,20]. Generally speaking, the MBE-grown GeSn thin films hold advantages on high Sn concentration [21] while the CVD grown ones show high optical property [7,14,15,16,17,22,23,24]. So far, to the best of our knowledge, all GeSn lasers were grown by CVD [7,14,15,16,17,25,26]. The causes for the difference in the optical properties of GeSn alloys grown by MBE and CVD are not fully clear. Structural property is usually a fatal factor on optical property. Crystalline fluctuations on a microscopic scale, line defects like dislocations and point defects such as vacancies can all result in degradation of optical property.

In this work, we investigate the structural property differences of GeSn alloys with different Sn concentrations and thicknesses. The structural properties of two groups of GeSn samples grown by MBE and CVD, respectively, are investigated through high resolution X-ray diffraction (XRD) and cross-sectional transmission electron microscopy (XTEM). We further study the differences of optical properties of GeSn samples grown by MBE and CVD and their connections with the structural properties. 

## 2. Materials and Methods 

The first group of six GeSn samples (M1-M6) were grown on Ge (001) substrate by MBE with Sn concentrations varying from 3.4% to 7.6%. The Ge (001) substrate was firstly deoxidized at 550 °C for 30 min. Secondly, a Ge buffer layer of about 100 nm was grown on the Ge (001) substrate at 500 °C. Thirdly, the temperature was lowered to 200 °C and the GeSn layer was grown. The detailed growth procedure was described elsewhere [27]. Another group of three GeSn samples (C1-C3) were grown by CVD with the Sn concentrations of 6.8%, 7.6% and 10.4%, respectively. A relaxed Ge virtual substrate (VS) of about 2.5 μm was firstly grown on the Si (001) substrate at 750 °C and 20 Torr. Secondly, to reduce the dislocation densities in the Ge-VS, the Ge-VS was annealed at 890 °C for 5 min and then the annealing was repeated for three times. Thirdly, the GeSn layer was deposited under a fixed precursor partial pressure ratio for the digermane (Ge_2_H_6_): tintetrachloride (SnCl_4_) = 220. The Sn content was controlled by adjusting the growth temperature. The detailed growth methods for Ge-VS and the GeSn layer can be found in [28] and [29], respectively. 

**Figure 1:**
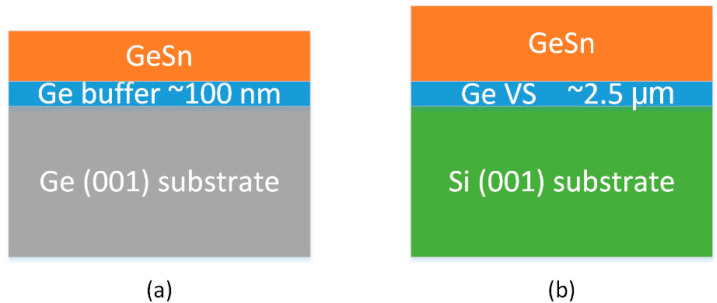
The sample structures of GeSn thin films grown by MBE (**a**) and CVD (**b**), respectively.

Figure 1 shows the sample structures grown by MBE (a) and CVD (b), respectively. The six GeSn samples grown by MBE are of different thicknesses and different Sn concentrations. For the CVD grown GeSn thin films, the three samples are grown on fully relaxed Ge VS. Threading dislocations were found in the annealed Ge VS, with the density of about 10^7^ cm^−2^ [28]. If the dislocation densities in the Ge VS is significantly reduced or the GeSn thin films are directly grown on Ge substrate by CVD, the structural and optical properties of the GeSn thin films might be further improved. The thicknesses and Sn contents of all the samples can be found in Table 1.

Crystallographic properties were measured by high resolution XRD with an X’Pert PRO diffractometer (Philips/Panalytical, Almelo, The Netherlands). An X-ray source with Cu Kα1 (*λ* = 1.54 Å) was used and traveled to the GeSn samples and then diffracted. There were two different optical paths (Optics1 and Optics2) for the detections. In Optics1, the diffracted beam directly traveled to the detector. In Optics2, the diffracted beam traveled to a three-crystal Ge (002) analyzer and then reached the detector. The thicknesses of GeSn layers were inferred from the calibrated growth rate and the XTEM data. The Sn concentrations were extracted from the Pendellösung interference fringes from symmetric (004) rocking curve (*ω*-2*θ*) scans. When getting the (004) *ω*-2*θ* rocking curves, Optics1 was used. The applied voltage and current were 30 KV and 25 mA, respectively. The angle step in the *ω*-2*θ* direction were both 0.0005° while the time for each step was 0.5 s. Then the broadenings of the GeSn epilayer features were investigated by two-dimensional reciprocal space mapping (2DRSM) around the asymmetric (224) reflections. When collecting the 2DRSM, Optics2 was used. The applied voltage and current were 40 KV and 40 mA, respectively. The angle steps along the *ω* direction and the *ω*-2*θ* direction were both 0.002° while the times for each step were 1.2 s. Furthermore, the structural properties of the GeSn layer were analyzed through XTEM using a Tecnai G2 F20 system (FEI, Hillsbro, OR, USA).

## 3. Results and Discussion

The mismatch dislocation density is related to the critical thickness of the epilayer. According to People-Bean’s model, the critical thickness *h_c_* is:

(1) $$h_{c}=\frac{1}{16\pi \sqrt{2}}\left(\frac{1-v}{1+v}\right)\frac{b^{2}}{af^{2}}ln\frac{h_{c}}{b}$$

where *v* is the Poisson’s ratio (*v*~0.26) [30], *a* is the lattice constant of Ge_1−x_Sn_x_, *f* is the misfit between Ge and Ge_1−x_Sn_x_ and *b* is the Burgers vector (*b*~0.4 nm) [31]. The Sn content, thickness and degree of strain relaxation and the calculated critical thickness of the GeSn layers are listed in Table 1.

The degree of strain relaxations for M1-M4 are 0, indicating the GeSn layers in M1-M4 are fully strained to the Ge substrate. The degree of strain relaxations for M5 and M6 are 7.2% and 14.2%, respectively, while for C1-C3 are 72.5%–82.7%, indicating that the GeSn layers in M5-M6 are partially relaxed to the Ge substrate and the GeSn layers in C1-C3 are almost fully relaxed to the Ge-VS.

For all the GeSn samples grown by CVD, the thickness exceeds the corresponding critical thickness and the relaxation rates are high. For the ones grown by MBE, the thickness of M1–M3 with low Sn content is below the critical thickness without strain relaxation, while the thickness of M4–M6 with high Sn content is above the critical thickness, experimentally. For M4, although its thickness exceeds the calculated critical thickness, the measured relaxation rate is still 0, indicating an underestimation by the theoretical model. For the GeSn samples with the same Sn content of 7.6%, the CVD grown one (C2) shows much higher relaxation rate than that of the MBE grown one (M6). The difference is probably caused by the much thicker thickness of C2 than M6.

**Figure 2:**
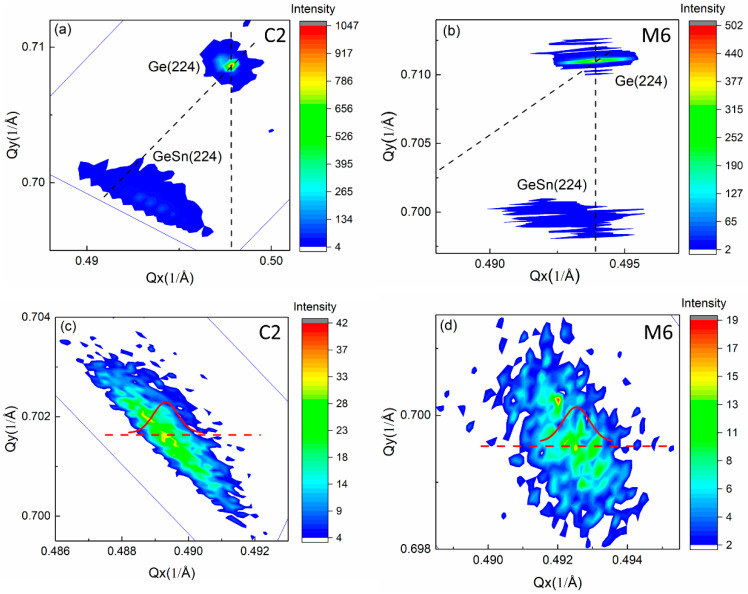
The 2DRSMs of two representative GeSn samples of C2 (left column) and M6 (right column), respectively. (**a**,**b**) are the 2DRSMs including the peaks of Ge layer and GeSn epi-layer. The vertical and diagonal black dotted lines represent the lattices pseudomorphically and fully relaxed to the substrate, respectively. (**c**,**d**) show the 2DRSMs of only the GeSn epilayer. The red dashed lines and solid curves in the figures indicate the cross-sections where and how the intensity is extracted and fitted by Gaussian equation, in the directions of parallel to the surface.

The structural properties of all the GeSn samples are further analyzed by 2DRSM around the asymmetric (224) diffractions by high resolution XRD. All the mappings are collected using *ω*-2*θ* scans offset along the *ω* directions. The 2DRSMs of C2 and M6 are shown in Figure 2. Figure 2a,b show the features of Ge layer and GeSn layer, while Figure 2c,d show the features of GeSn layer. The broadening of the diffraction peaks in 2DRSM in different directions are caused by different origins. It is commonly accepted that the broadenings in the *ω* direction, along the *ω*-2*θ* direction and parallel to the surface are due to the wafer curvature and/or mismatch dislocations, the variation in *d*-spacing through the layer and the lateral incoherence, respectively [32].

**Figure 3:**
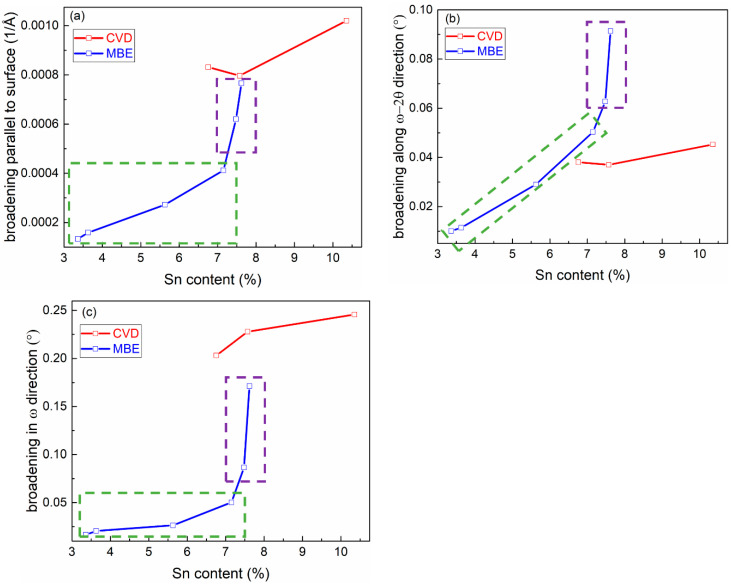
The broadening of the GeSn epilayer features parallel to the surface (**a**), along the *ω*-2*θ* direction (**b**) and in the *ω* direction (**c**). The red and blue curves represent the samples grown by CVD and MBE, respectively. The green and purple dotted square frame marked samples M1-M4 and M5-M6, of which the GeSn layers are fully strained and partially relaxed to Ge substrate, respectively. The CVD grown samples C1-C3 are almost fully relaxed to Ge-VS.

Figure 3 shows the broadenings of GeSn layers parallel to the surface (a), along the *ω*-2*θ* direction (b) and in the *ω* direction (c). The different broadening data are extracted as follows. The 2DRSM is plotted in *Qx* and *Qy* axes, as shown in Figure 2c,d. The diffraction peak is auto selected by the “X’Pert Epitaxy’ software. The diffraction intensity data on the line parallel to the *Qx* axis and cross the diffraction peak are extracted. The data are then fitted by a Gaussian equation. The full with at half maxima (FWHM) of the fitted Gaussian curve is extracted to be the broadenings parallel to the surface. Then the diffraction intensity is plotted in the axes of *ω*-2*θ* scan axis and the *ω* scan axis. The diffraction peak is also auto selected. Two sets of data on the line parallel to the *ω*-2*θ* scan axis crossing the diffraction peak and parallel to the *ω* scan axis crossing the diffraction peak are extracted, respectively. The FWHM of the Gaussian fitting to the two sets of data are the broadenings along the *ω*-2*θ* direction and in the *ω* direction, respectively.

**Figure 4:**
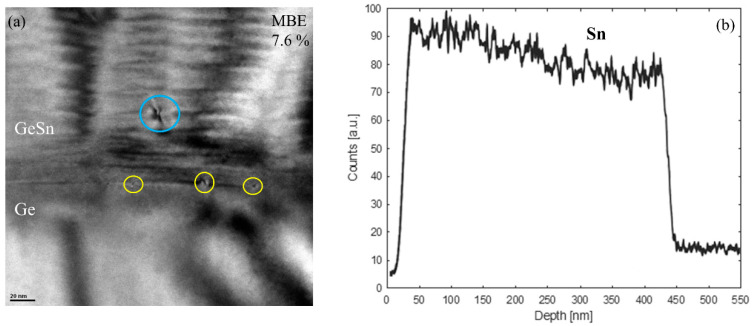
(**a**) XTEM and (**b**) the energy dispersive spectroscopy (EDX) of the GeSn sample with the Sn concentration of 7.6% grown by MBE. The EDX is collected from the surface of GeSn layer down to the Ge layer.

GeSn layers in M1–M4, M5, M6 and C1–C3 are fully strained, partially relaxed and almost fully relaxed to Ge substrate and (or) Ge-VS, respectively. The broadenings parallel to the surface of the GeSn epilayer feature of the M1-M6 are found monotonically increase with the Sn concentrations varying from 3.4% to 7.6%, as shown in Figure 3a. It sharply increases when the Sn concentration is above 7%, where the strain of GeSn layer starts to relax. C1-C3 show larger broadenings than M1–M6, indicating a worse lateral incoherence in C1–C3. This is mostly due to the fact that C1–C3 have large amount of edge dislocations concentrated at the GeSn/Ge interface causing local strain variation. However, the broadenings along the *ω*-2*θ* direction (seen in Figure 3b) of the GeSn feature of C1–C3 are smaller than that of M4–M6. It implies that the Sn distribution is quite nonuniform in the samples with high Sn content grown by MBE, which is in consistence with the EDX line-scan result shown in Figure 4b. A gradual increase of Sn concentration is observed from the bottom to the top of the GeSn thin film.

**Figure A1:**
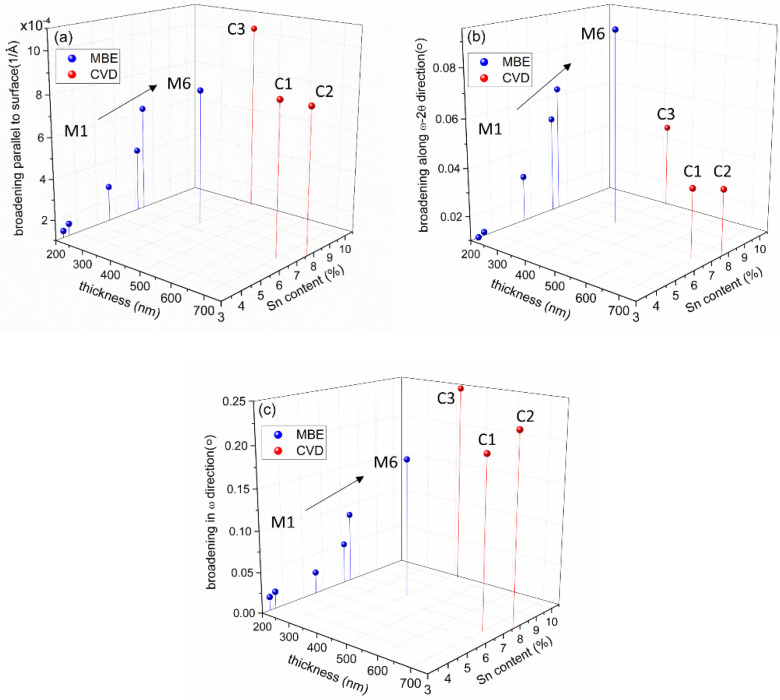
3D plot of the broadenings of the GeSn epilayer features parallel to the surface (**a**), along the *ω*-2*θ* direction (**b**) and in the ω direction (**c**). The blue and red balls represent the samples grown by MBE and CVD, respectively.

The broadening in the *ω* direction shows a similar trend to the broadening parallel to the surface. Since the thickness of the GeSn epilayers is very small compared with that of the Ge substrates or Ge-VS, the wafer curvature caused by strain in the GeSn layer is negligible. Therefore, dislocation is the most probable reason for the *ω* broadening. When the Sn concentration is above 7%, the density of the mismatch dislocations significantly increases indicating that the critical thickness of strain relaxation is reached. The *ω*-broadenings of C1–C3 are larger than that of M1–M6, due to larger relaxations of C1–C3. However, the strain relaxation through dislocation process is complicatedly associated to lattice mismatch, film thickness, type of dislocations, etc. A three-dimensional plot of the variations of the broadenings with different Sn contents and thicknesses can be found in Figure A1 in the ‘Appendix A’ section. A more intensive analysis is as follows.

Figure 4a shows the XTEM results of the M6. The blue circle is the threading part of 60° dislocations. Yellow circles are the edge dislocations or the misfit part of 60° dislocations. For the CVD grown GeSn samples, mostly pure edge dislocations together with small amount of 60° dislocations bending into the Ge layer were observed in TEM measurements, as shown in reference [7]. Figure 4b shows the EDX line-scan result of Sn content in M6. Sn content increases from the bottom of the GeSn layer to the surface, indicating a segregation of Sn atoms. Due to the low thermal equilibrium solid solubility of Sn in Ge, Sn segregation is a big challenge for the GeSn growth [27]. The nonuniform distribution of Sn atoms in M6 explains the large broadenings along the *ω*-2*θ* direction measured by the 2DRSM.

The mismatch dislocation density *ρ* is calculated as follows, according to Hu’s expression [33]:

(2) $$\rho =\frac{f}{bsin\theta sin\emptyset }\left(1-\frac{h_{c}}{h}\right)$$

In the above expression, *θ* is the angle between the dislocation line and the Burgers vector and *ϕ* is the angle between the slip plane and the interface. In GeSn systems, there are basically two types of dislocations, 60° and pure edge dislocations [34]. For 60° dislocations, *θ* is 60° and the slip plane is the (111) plane, so *ϕ* is 54.7°. For edge dislocations, *θ* is 90° and the slip plane is the (010) plane, so *ϕ* is 90°. Considering the relaxation rate measured by XRD, the contents in the bracket in expression (2) could be equivalent to the degree of strain relaxation (*R*), so the mismatch dislocation density is estimated as follows:

(3) $$\rho =\frac{fR}{bsin\theta sin\emptyset }$$

**Figure 5:**
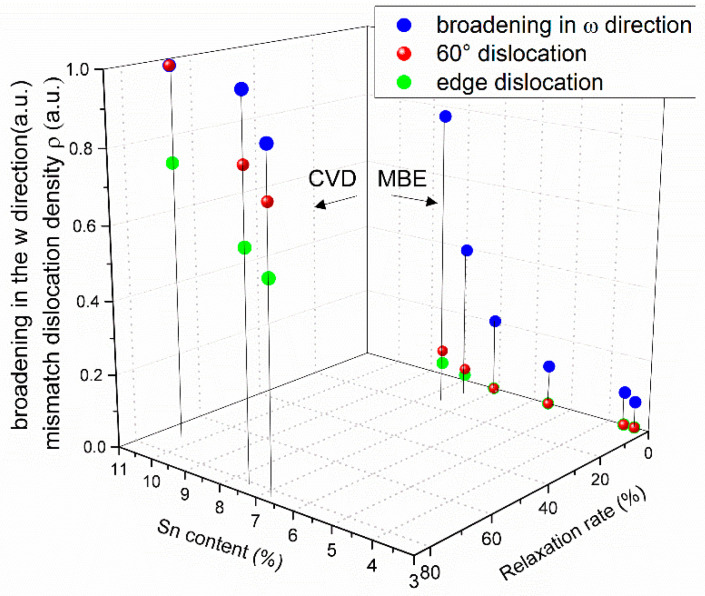
Normalized broadening in the *ω* direction (blue), and the dislocation density calculated based on all 60° dislocation (red) and all edge dislocation (green) assumption with different Sn contents and relaxations.

**Figure A2:**
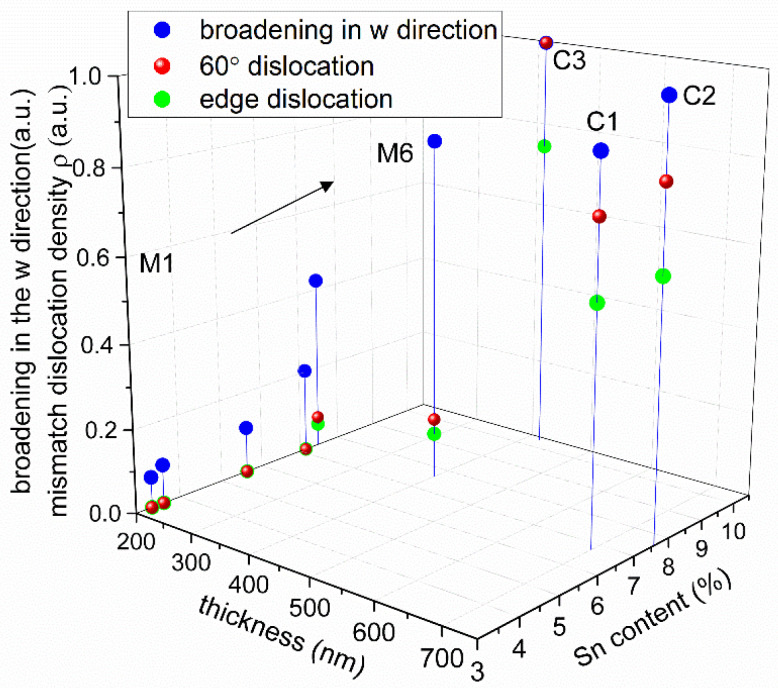
Normalized broadening in the *ω* direction (blue), and the dislocation density calculated based on all 60° dislocation (red) and all edge dislocation (green) assumption with different thicknesses and Sn contents.

The broadening in the *ω* direction (blue) and the dislocation density based on all 60° (red) and all edge dislocation (green) assumptions of the samples are shown in Figure 5. A three-dimensional plot of the variations of the broadenings and dislocation densities with different Sn contents and thicknesses can be found in Figure A2 in the in ‘Appendix A’ section. The dislocation density and the broadening in the *ω* direction are both normalized and share the *z* axis. The real dislocation density should lie between the two extremes, while the data points for C1–C3 would be fairly close to the pure edge dislocation case. The calculated dislocation density of both types of C1–C3 are significantly larger than that of M1–M6, due to the larger mismatch, thickness and relaxation. For C1–C3, a consistent trend is found for the *ω*-broadening and the dislocation density with the Sn content. While a large discrepancy is observed for the M1–M6. Firstly, for M1–M4 who are fully strained to the Ge substrate, considerable *ω*-broadening exists. Secondly, although the *ω*-broadening follows the calculated dislocation density for M5, M6, the *ω*-broadening is much larger compared with the trend of C1–C3. With the exclusion of the possible reasons of wafer curvature and peak broadening due to very thin film, other factors should exist, causing the broadening in the *ω* direction. High density of point defects might be one possible reason.

The CVD grown samples discussed above show strong photoluminescence (PL) at room temperature [29], while the MBE-grown ones show no PL. It is always complicated to relate the optical properties of semiconductors to structural properties. The commonly believed major factors are dislocation density, point defect density, overall crystalline quality, etc. Through the analysis above, the CVD grown samples on Ge-VS have much larger density of dislocations than the MBE-grown ones. However, the dislocations in the CVD grown samples distributes majorly at the GeSn/Ge interface or inside the Ge buffer layer, without threading dislocations extending upwards in the GeSn layer. To the contrary, MBE-grown ones have considerable amount of threading dislocations in the GeSn layer although the overall dislocation density is lower. The MBE-grown samples show no PL, but when we etched the GeSn films into suspended cantilever microstructures to relax the strain, week PL was obtained [35]. For other lattice properties, such as lateral incoherence, the CVD grown samples show no advantages. Although MBE-grown GeSn shows a higher compositional nonuniformity, it is commonly believed not an important factor for optical property. Local states may even enhance photoluminescence. Another important factor, which can’t be directly measured by XRD, is the point defect density. The point defects, such as vacancies, can act as nonradiative recombination centers. The CVD process to grow GeSn uses a much higher growth temperature than that by MBE. It is generally true that the higher growth temperature the lower point defect density. The “hidden” factor who causes the broadening in *ω* direction for the MBE samples could be an important factor. To sum up, the high density of threading dislocation and point defects could be the most probable reasons for the worse optical property of MBE-grown GeSn than the CVD grown ones.

The major limitation of this experiment is that the substrates and the sample structure of the MBE and the CVD grown samples are different, leading to difficulty in direct comparison.

## 4. Conclusions

In this paper, structural properties of GeSn alloys with different Sn contents and thicknesses grown by MBE (low relaxation) and CVD (high relaxation) are analyzed through XRD and XTEM. The GeSn layers fully strained to Ge substrate shows the best structural properties. The structural properties, such as lateral incoherence and the density of mismatch dislocations of GeSn layers partially relaxed to Ge substrate are found better than that of GeSn layers almost fully relaxed to Ge-VS. However, the GeSn alloys grown by MBE has higher threading dislocation density than that grown by CVD. Meanwhile, GeSn layers partially relaxed to Ge substrate grown by MBE show larger compositional nonuniformity than that of GeSn layers almost fully relaxed to Ge-VS grown by CVD. Point defects and the threading dislocations are two possible reasons for the poor optical property of the GeSn grown by MBE.

## Figures and Tables

**Table 1:** The Sn content, thickness, calculated critical thickness and degree of strain relaxation of the GeSn epilayers grown by MBE and CVD.

	Sample Number	Sn Content (%)	Thickness (nm)	Critical Thickness (nm)	Relaxation (%)
MBE	M1	3.4	200	691.5	0
	M2	3.6	200	578	0
	M3	5.6	200	205.5	0
	M4	7.2	200	115.4	0
	M5	7.5	200	103.3	7.2
	M6	7.6	400	98.7	14.2
CVD	C1	6.8	700	132.3	82.7
	C2	7.6	750	98.7	80.7
	C3	10.4	420	45.8	72.5

## Appendix A

Figure A1 shows the variations of the broadenings of the GeSn epilayer features with different Sn contents and thicknesses. The tendency for the variations of the broadenings parallel to the surface, along the *ω*-2*θ* direction and in the ω direction for M1–M6 are similar. The thicknesses for M1–M5 are 200 nm, the broadenings increases with the Sn contents. For M5 and M6, the Sn contents are 7.5% and 7.6%, almost the same, the thicknesses are 200 nm and 400 nm, respectively. The larger broadenings of M6 than that of M5 is mainly due to the increases of the thickness. In Figure A1a,c, C1–C3 show larger broadenings parallel to the surface and in the ω direction than M1–M6, respectively. It indicates a worse lateral incoherence and larger amount of mismatch dislocations in C1–C3.

In Figure A1b, C1–C3 show smaller broadenings along the *ω*-2*θ* direction than M5,M6, indicating a nonuniform distribution of Sn atoms in the MBE samples with high Sn content, which is in consistent with the EDX line-scan result shown in Figure 4b.

Figure A2 shows the variations of the broadening in the *ω* direction and the dislocation density based on all 60 degree and all edge dislocation assumptions of the samples with different thicknesses and Sn contents. The dislocation density and the broadening in the *ω* direction are both normalized and share the *z* axis.

## References

[1] Mack C.A. (2011). Fifty years of Moore’s law. IEEE Trans. Semicond. Manuf..

[2] Courtland R. (2016). Transistors could stop shrinking in 2021. IEEE Spectr..

[3] Thomson D., Zilkie A., Bowers J.E., Komljenovic T., Reed G.T., Vivien L., Marris-Morini D., Cassan É., Virot L., Fédéli J.-M. (2016). Roadmap on silicon photonics. J. Opt..

[4] Soref R. (2006). The Past, Present, and Future of Silicon Photonics. IEEE J. Sel. Top. Quantum Electron..

[5] Bowers J.E., Komljenovic T., Davenport M., Hulme J., Liu A.Y., Santis C.T., Spott A., Srinivasan S., Stanton E.J., Zhang C. Recent advances in silicon photonic integrated circuits. Proceedings of the Next-Generation Optical Communication: Components, Sub-Systems, and Systems V.

[6] Gupta S., Magyari-Kope B., Nishi Y., Saraswat K.C. (2013). Achieving direct band gap in germanium through integration of Sn alloying and external strain. J. Appl. Phys..

[7] Wirths S., Geiger R., Driesch N.V.D., Mussler G., Stoica T., Mantl S., Ikonić Z., Luysberg M., Chiussi S., Hartmann J.M. (2015). Lasing in direct-bandgap GeSn alloy grown on Si. Nat. Photonics.

[8] De Kersauson M., El Kurdi M., David S., Checoury X., Fishman G., Sauvage S., Jakomin R., Beaudoin G., Sagnes I., Boucaud P. (2011). Optical gain in single tensile-strained germanium photonic wire. Opt. Express.

[9] Süess M.J., Geiger R., Minamisawa R., Schiefler G., Frigerio J., Chrastina D., Isella G., Spolenak R., Faist J., Sigg H. (2013). Analysis of enhanced light emission from highly strained germanium microbridges. Nat. Photonics.

[10] Sau J.D., Cohen M.L. (2007). Possibility of increased mobility in Ge-Sn alloy system. Phys. Rev. B.

[11] Maeda T., Wipakorn J., Hiroyuki H., Noriyuki U., Jean-Pierre L., Ruben L. Junctionless GeSn pMOSFETs on Si (111) by solid phase epitaxy. Proceedings of the International Conference on Silicon epitaxy and Heterostructures (ICSI-8).

[12] Guo P., Han G., Gong X., Liu B., Yang Y., Wang W., Zhou Q., Pan J., Zhang Z., Tok E.S. (2013). Ge0.97Sn0.03 p-channel metal-oxide-semiconductor field-effect transistors: Impact of Si surface passivation layer thickness and post metal annealing. J. Appl. Phys..

[13] Maeda T., Jevasuwan W., Hattori H., Uchida N., Miura S., Tanaka M., Santos N.D.M., Vantomme A., Locquet J.-P., Lieten R.R. (2015). Ultrathin GeSn p-channel MOSFETs grown directly on Si(111) substrate using solid phase epitaxy. Jpn. J. Appl. Phys..

[14] Al-Kabi S., Ghetmiri1 S.A., Margetis J., Pham T., Zhou Y., Dou W., Collier B., Quinde R., Du W., Mosleh A. (2016). An optically pumped 2.5 μm GeSn laser on Si operating at 110 K. Appl. Phys. Lett..

[15] Stange D., Wirths S., Geiger R., Schulte-Braucks C., Marzban B., Driesch N.V.D., Mussler G., Zabel T., Stoica T., Hartmann J.-M. (2016). Optically Pumped GeSn Microdisk Lasers on Si. ACS Photonics.

[16] Stange D., Driesch N.V.D., Zabel T., Armand-Pilon F., Rainko D., Marzban B., Zaumseil P., Hartmann J.-M., Ikonic Z., Capellini G. (2018). GeSn/SiGeSn Heterostructure and Multi Quantum Well Lasers. ACS Photonics.

[17] Reboud V., Gassenq A., Pauc N., Aubin J., Milord L., Thai Q.M., Bertrand M., Guilloy K., Rouchon D., Rothman J. (2017). Optically pumped GeSn micro-disks with 16% Sn lasing at 3.1 um up to 180K. Appl. Phys. Lett..

[18] Kasper E., Werner J., Oehme M., Escoubas S., Burle N., Schulze J. (2012). Growth of silicon based germanium tin alloys. Thin Solid Films.

[19] Wirths S., Buca D., Mantl S. (2016). Si–Ge–Sn alloys: From growth to applications. Prog. Cryst. Growth Charact. Mater..

[20] Zaima S., Nakatsuka O., Taoka N., Kurosawa M., Takeuchi W., Sakashita M. (2015). Growth and applications of GeSn-related group-IV semiconductor materials. Sci. Technol. Adv. Mater..

[21] Oehme M., Kostecki K., Schmid M., Oliveira F., Kasper E., Schulze J. (2014). Epitaxial growth of strained and unstrained GeSn alloys up to 25% Sn. Thin Solid Films.

[22] Mathews J., Beeler R.T., Tolle J., Xu C., Roucka R., Kouvetakis J., Menéndez J. (2010). Direct-gap photoluminescence with tunable emission wavelength in Ge_1−y_ Sn_y_ alloys on silicon. Appl. Phys. Lett..

[23] Margetis J., Mosleh A., Al-Kabi S., Ghetmiri S., Du W., Dou W., Benamara M., Li B., Mortazavi M., Naseem H. (2017). Study of low-defect and strain-relaxed GeSn growth via reduced pressure CVD in H 2 and N 2 carrier gas. J. Cryst. Growth.

[24] Ghetmiri S.A., Du W., Margetis J., Mosleh A., Cousar L., Conley B.R., Domulevicz L., Nazzal A., Sun G., Soref R. (2014). Direct-bandgap GeSn grown on silicon with 2230 nm photoluminescence. Appl. Phys. Lett..

[25] Margetis J., Al-Kabi S., Du W., Dou W., Zhou Y., Pham T., Grant P., Ghetmiri S., Mosleh A., Li B. (2017). Si-based GeSn lasers with wavelength coverage of 2–3 μm and operating temperatures up to 180 K. ACS Photonics.

[26] Thai Q.M., Pauc N., Aubin J., Bertrand M., Chrétien J., Delaye V., Chelnokov A., Hartmann J., Reboud V., Calvo V. (2018). GeSn heterostructure micro-disk laser operating at 230 K. Opt. Express.

[27] Zhang Z.P., Song Y., Zhu Z.Y.S., Han Y., Chen Q.M., Li Y.Y., Zhang L.Y., Wang S.M. (2017). Structural properties of GeSn thin films grown by molecular beam epitaxy. AIP Adv..

[28] Hartmann J.M., Abbadie A., Cherkashin N., Grampeix H., Clavelier L. (2009). Epitaxial growth of Ge thick layers on nominal and 6° off Si(0 0 1); Ge surface passivation by Si. Semicond. Sci. Technol..

[29] Driesch N.V.D., Stange D., Wirths S., Mussler G., Holländer B., Ikonic Z., Hartmann J.M., Stoica T., Mantl S., Grützmacher D. (2015). Direct Bandgap Group IV Epitaxy on Si for Laser Applications. Chem. Mater..

[30] http://www.ioffe.ru/SVA/NSM/Semicond/Ge/mechanic.html.

[31] People R., Bean J. (1986). Erratum: Calculation of critical layer thickness versus lattice mismatch for Ge_x_Si_1−x/_Si strained-layer heterostructures [Appl. Phys. Lett. 47, 322 (1985)]. Appl. Phys. Lett..

[32] Moram M.A., Vickers M.E. (2009). X-ray diffraction of III-nitrides. Rep. Prog. Phys..

[33] Hu S.M. (1991). Misfit dislocations and critical thickness of heteroepitaxy. J. Appl. Phys..

[34] Dou W., Benamara M., Mosleh A., Margetis J., Grant P., Zhou Y., Al-Kabi S., Du W., Tolle J., Li B. (2018). Investigation of GeSn Strain Relaxation and Spontaneous Composition Gradient for Low-Defect and High-Sn Alloy Growth. Sci. Rep..

[35] Yi H., Song Y., Xiren C., Zhang Z., Liu J., Li Y.Y., Zhu Z., Huang H., Shao J., Wang S. (2018). Abnormal strain in suspended GeSn microstructures. Mater. Res. Exp..

